# Draft genome assemblies of 35 bacteria isolated from hibernating arctic ground squirrels

**DOI:** 10.1128/mra.00972-24

**Published:** 2025-01-27

**Authors:** Heidi McKee, Logan Mullen, Devin M. Drown, Khrystyne N. Duddleston

**Affiliations:** 1 Department of Biological Sciences, University of Alaska Anchorage, Anchorage, Alaska, USA; 2 Institute of Arctic Biology, University of Alaska Fairbanks, Fairbanks, Alaska, USA; 3 Department of Biology and Wildlife, University of Alaska Fairbanks, Fairbanks, Alaska, USA; University of Maryland School of Medicine, Baltimore, Maryland, USA

**Keywords:** host-microbe, beneficial, environmental microbiology, Nanopore

## Abstract

Arctic ground squirrels (*Urocitellus parryii*) hibernate for several months without eating or drinking yet suffer no disuse atrophy. We are investigating the potential contributions of gut microorganisms to host nitrogen homeostasis, and here, we describe the genome assemblies of 35 isolated bacteria collected from gastrointestinal material and sequenced using Nanopore technology.

## ANNOUNCEMENT

Arctic ground squirrels (*Urocitellus parryii*) hibernate for 7–9 months of the year, during which they neither eat nor drink and regulate their body temperature as low as −2.9°C (1). Remarkably, they lose relatively little lean mass during this period, emerging fully functional and absent disuse atrophy (2). Recent evidence suggests that gut microbes may contribute to nitrogen recycling during hibernation (3).

Fecal pellets and cecum contents were collected from euthanized hibernating arctic ground squirrels (UAA-IACUC #1428231) and stored in brucella broth and 20% glycerol at −80°C. Thawed, homogenized samples were diluted in phosphate-buffered saline (PBS), spread plated onto a diversity of media, and incubated under aerobic, microaerophilic, or anaerobic headspace at 28°C or 37°C (Table 1) (4). Subsets of samples were heated (65°C, 20 min) prior to plating to target endospore formers. Colonies with unique morphology were sub-cultured 3× onto Brain Heart Infusion (BHI) media to obtain pure cultures. Isolates were grown in BHI broth and then stored in an equal part of 50% sterile glycerol at −80°C. The culture collection is housed in the Advanced Instrumentation for Microbiome Studies (AIMS) Core Lab Facility at the University of Alaska Anchorage.

**TABLE 1:** Bacterial isolate accession numbers, taxonomic assignment, and sample material/culture conditions summary

Isolate	Biosample accession	GenBank accession	SRA accession	Whole genome similar (reference and representative sequence)			Squirrel	Type
				Closest match	p-value	Distance		
AR001	SAMN41327269	CP160000	SRR29418665	Bacillus infantis NRRL B-14911	0	0.0172	19–33	Fecal
AR002	SAMN41327270	CP159999	SRR29418664	Paenibacillus xylanexendens str. PAMC 22703	0	0.0449	19–33	Fecal
AR003	SAMN41327271	JBFDTO000000000	SRR29418653	Staphylococcus capitis str. AATZJ	0	0.0002	19–33	Fecal
AR005	SAMN41327272	JBFDTN000000000	SRR29418642	Streptococcus sp. HTS2	7.29E-106	0.1477	19–33	Fecal
AN401	SAMN41327273	JBDOIQ000000000	SRR29418635	Erisipelatoclostridium ramosum str. FDAARGOS_1105	0	0.0087	19–26	Fecal
AN402	SAMN41327274	JBFDTM000000000	SRR29418634	Enterococcus faecalis T5	0	0.0162	19–26	Fecal
AN404	SAMN41327275	JBFDTL000000000	SRR29418633	Staphylococcus epidermidis str. ATCC 14990	0	0.0145	19–26	Fecal
AN405	SAMN41327276	CP159998	SRR29418632	Intestimonas butyriciproducens str. 27-5-10	0	0.0191	19–26	Fecal
AN406	SAMN41327277	JBDOIP000000000	SRR29418631	[Clostridium] innocuum str. ATCC 14501	0	0.0121	19–26	Fecal
AR505	SAMN41327278	CP159997	SRR29418630	Bacillus safensis str. PgKB20	0	0.0349	19–26	Fecal
AR506	SAMN41327279	JBDOIO000000000	SRR29418663	Paenibacillus illinoisensis NBRC 15959	0	0.0748	19–26	Fecal
AR507	SAMN41327280	CP159996	SRR29418662	Terrabacillus aidingensis str. MP602	0	0.0932	19–26	Fecal
AR509	SAMN41327281	JBDOIN000000000	SRR29418661	Paenibacillus amylolyticus str. FSL H7-0692	0	0.0446	19–26	Fecal
AR510	SAMN41327282	JBGFSU000000000	SRR29418660	Bacillus gibsonii str. KCCM 41407	0	0.0261	19–26	Fecal
M501	SAMN41327283	CP159995	SRR29418659	Bacillus safensis str. PgKB20	0	0.0349	19–26	Fecal
M502	SAMN41327284	JBGFST000000000	SRR29418658	Bacillus safensis str. PgKB20	0	0.0373	19–26	Fecal
AR601	SAMN41327285	CP159994	SRR29418657	Escherichia coli str.K-12 substr.MG1655	0	0.0295	19–26	Fecal
AN502	SAMN41327286	JBGFSP000000000	SRR29418656	Bacteriodes xylansolvens CL11T00C03	3.58E-172	0.1233	19–26	Fecal
AN1001	SAMN41327287	JBGFSO000000000	SRR29418655	Lactobacillus sp. P38	0	0.0444	19–11	Cecal
M1002	SAMN41327288	CP159993	SRR29418654	Bacillus amyloliquefaciens str. GKT04	0	0.0134	19–11	Cecal
M1003	SAMN41327289	JBDOIM000000000	SRR29418652	Bacillus megaterium str. 22–2	0	0.0293	19–11	Cecal
AN1005	SAMN41327290	JBGFQW000000000	SRR29418651	Niallia taxi MER 100	0	0.0583	19–11	Cecal
AN1007	SAMN41327291	CP159992	SRR29418650	Paenibacillus sp. BL9	9.14E-203	0.1158	19–11	Cecal
AN1009	SAMN41327292	CP159991	SRR29418649	Alloscardovia sp. GLD14/2	9.66E-17	0.2301	19–11	Cecal
AN1013	SAMN41327293	JBFDTK000000000	SRR29418648	Enterococcus hirae FDAARGOS_234	0	0.0108	19–11	Cecal
AN2001	SAMN41327294	JBDOIL000000000	SRR29418647	Escherichia coli str.K-12 substr.MG1655	0	0.0305	19–11	Cecal
AN2004	SAMN41327295	CP159990	SRR29418646	Ruminococcus sp. AT12	1.17E-27	0.2036	19–11	Cecal
AR3001	SAMN41327296	JBGFSS000000000	SRR29418645	Bacillus safensis str. PgKB20	0	0.0232	19–11	Cecal
AR3002	SAMN41327297	JBGFSR000000000	SRR29418644	[Brevibacterium] frigoritolerans JHS1	0	0.0289	19–11	Cecal
M3003	SAMN41327298	JBDOIK000000000	SRR29418643	Escherichia coli str.K-12 substr.MG1655	0	0.0306	19–11	Cecal
M3004	SAMN41327299	JBDOIJ000000000	SRR29418641	Paenibacillus amylolyticus str. FSL H7-0692	0	0.0457	19–11	Cecal
AR3005	SAMN41327300	JBDOII000000000	SRR29418640	Escherichia coli str.K-12 substr.MG1655	0	0.0305	19–11	Cecal
AR3006A	SAMN41327301	JBGFSQ000000000	SRR29418639	[Brevibacterium] frigoritolerans JHS1	0	0.0255	19–11	Cecal
AR3006B	SAMN41327302	JBFDTJ000000000	SRR29418638	Paenibacillus illinoisensis NBRC 15959	0	0.0417	19–11	Cecal
AR004	SAMN41327304	CP159989	SRR29418636	Actinomyces sp. zg-235	0	0.0552	19–33	Fecal

^*a*^ Complete Table 1 with sequencing and assembly information and culture conditions: DOI 10.6084/m9.figshare.28250759

Isolates were selected for long-read full genome sequencing based on urease activity and/or the absence of a species-level match NCBI reference sequence to the trimmed (5) 16S rRNA sanger sequence (Azenta). We grew bacteria in BHI broth agar (Table 1), then pelleted and washed the cells 3× in 1 mL sterile PBS (10,000 × *g*). DNA was extracted from either fresh or previously frozen cell pellets (−80°C) using the Qiagen DNeasy UltraClean Microbial Kit (Hilden, Germany), then stored (−20°C) until sequencing. Samples were prepared as a multiplex sequencing rapid library (SQK-RBK114-24) and sequenced on a GridION using R10.4.1 flow cells (IDs: run1, FAX55814; run2, FAX55831). We base called, filtered by quality score (*Q*-score ≥ 10), and demultiplexed the raw data using Guppy version 6.5.7 (dna_r10.4.1_e8.2_400bps_sup@v4.2.0). Run 1 generated 12.91 Gb in 6.1 M reads with a read *N*_50_ of 3.85 kb. Run 2 generated 15.35 Gb in 6.4 M reads with a read *N*_50_ of 4.55 kb. FASTQ data were processed into polished assemblies using the EPI2ME Bacterial Genomes workflow version 2.6.1 (https://github.com/epi2me-labs/wf-metagenomics) that includes Flye version 2.9.2 (6) for assembly and Medaka version 1.9.1 (https://github.com/nanoporetech/medaka) for error correction. Default parameters were used except where otherwise noted. Table 1 summarize our complete genomes with assembly statistics (including completeness and contamination) from BV-BRC Comprehensive Genome Analysis Service. Taxonomic assignment of full genomes reflects the closest match to the reference sequences database when searching for similar genomes with BV-BRC similar genome finder tool [(7); Table 1].

The whole genomes deposited in GenBank were annotated with PGAP (8) as part of the submission pipeline (Table 1). Data for this project are stored under BioProject PRJNA1107577.

## Data Availability

This Whole Genome Shotgun project has been deposited in GenBank under the accession PRJNA1107577.
